# *Mycobacterium chimaera* infections associated with heater-cooler units (HCU): closing another loophole in patient safety

**DOI:** 10.2807/1560-7917.ES.2016.21.46.30397

**Published:** 2016-11-17

**Authors:** Marc J. Struelens, Diamantis Plachouras

**Affiliations:** 1 European Centre for Disease Prevention and Control (ECDC), Stockholm, Sweden

**Keywords:** Mycobacterium chimaera, waterborne infections, device-associated infections, patient safety

In 2011, invasive cardiovascular and disseminated infections by a slowly-growing non-tuberculous mycobacterium, *Mycobacterium chimaera,* were detected in patients who had undergone cardiothoracic surgery in Switzerland. *M. chimaera* was subsequently detected in the water tanks of heater-cooler units (HCUs) used to regulate the temperature of patients’ blood in the cardiopulmonary bypass circuit, and in air samples from the operating room when the HCUs were running [1]. This report led investigators in other countries to look for similar cases among cardiothoracic surgery patients exposed to such devices. From 2014 onwards up to April 2015, cases of invasive cardiovascular infection by *M. chimaera* potentially linked to HCUs were consecutively detected in the Netherlands, Germany and the United Kingdom (UK) [2] and hereafter in the United States (US) [3]. An epidemiological link with use of a specific model of HCUs, the 3T device (LivaNova, UK; formerly Sorin, Germany), was confirmed by the detection of *M. chimaera* in these devices across affected cardiothoracic surgery centres [4]. Observational and experimental studies showed that exhaust air from contaminated HCUs can transmit aerosols with *M. chimaera* to the operating field under ultraclean laminar air flow ventilation [5,6].

Environmental testing at the manufacturing site identified contamination with *M. chimaera* of water tanks of LivaNova/Sorin 3T HCUs, as well as of water from the pump assembly area of the facility [4]. In April 2016, the preliminary results of an analysis of the whole genome sequence of outbreak-related *M. chimaera* isolates showed ‘almost identical genome sequences’ among clinical isolates from patients in three European countries and environmental isolates from 3T devices in the affected hospitals and at the device manufacturing site. These findings supported the hypothesis of a common-source, multi-country outbreak related to intrinsic contamination of 3T devices manufactured before September 2014 [4]. Recently, a study of whole-genome sequences of clinical isolates from *M. chimaera* infected open-heart surgery patients and from HCUs from hospitals in Pennsylvania and Iowa, US, reportedly showed few single nucleotide polymorphism (SNP) differences between outbreak-related isolates as compared with hundred-fold larger SNP differences between outbreak-related isolates and an epidemiologically unrelated isolate [7]. However, whole-genome sequence data from the outbreak investigations in Europe and the US have not been published to date.

In this issue of *Eurosurveillance*, the first case of *M. chimaera* pleural infection in a lung transplant recipient from Australia is reported, together with results of environmental investigations that indicate frequent contamination with *M. chimaera* of HCU devices used in hospitals across Western Australia, suggesting that the outbreak extends beyond Europe and the US [8]. This report tests potential source hypotheses by whole-genome sequencing of clinical and environmental *M. chimaera* isolates. Of particular interest is the finding that the genomes of isolates from HCUs across four hospitals clustered in two groups, each composed of isolates differing by less than 17 SNPs. It remains to be seen whether these *M. chimaera* genotypes match those from HCUs in Europe and the US. Of note, a clinical isolate from the infected patient potentially exposed to one of the contaminated HCUs did not match environmental genotypes and showed over 600 SNPs differences from the isolates recovered from the devices. Although, in this case, the results were found sufficient to rule out the HCU as the source of infection, the authors recognise the limitation of their sampling method based on single colony genome analysis, which may have missed mixed-strain populations that were present in the tested samples. Furthermore, the whole-genome comparative analysis of a larger collection of *M. chimaera* isolates, including from sporadic infections and environmental reservoirs worldwide, is awaited. It should reveal the genetic population structure of *M. chimaera* and ascertain the extent of common source contamination of HCUs as well as the fraction of HCU-associated infections attributable to the 3T device. To the best of our knowledge, the sharing before publication of preliminary genome sequence data on this emerging pathogen through public repositories, as advocated for improving public health investigations of international epidemics [9,10], has not yet been implemented.

In a second study in this issue, the occurrence of *M. chimaera* infection associated with treatment by extracorporeal membrane oxygenation (ECMO) devices was explored in a retrospective descriptive clinical study combined with prospective environmental sampling at a German supra-regional ECMO centre [11]. ECMO also uses thermoregulatory devices and is regarded as a potential further source for *M. chimaera* infections in a group of severely ill and often immunocompromised patients. However, in contrast to HCUs used in cardio-thoracic surgery, ECMOs are air-tight and closed systems, plausibly precluding the release of aerosols. Contamination with *M. chimaera* of water tanks from ECMO thermoregulatory devices from two manufacturers was documented, but no room air contamination was found. No patients with *M. chimaera* infection linked to ECMO devices were identified during the period of intensive care. A limitation of this single-centre study is the relatively short patient follow-up. Further prospective studies should elucidate the clinical relevance, if any, of *M. chimaera* contamination of ECMO devices.

Recognising the health hazard associated with mycobacterial contamination of HCUs used in cardio-thoracic surgery, national authorities in Europe and the US have issued health alerts to surgical facilities. They call for increased vigilance, active surveillance and implementation of risk mitigation measures such as removal of the HCU from the operating room to a side room as well as implementation of the updated decontamination and cleaning protocol as provided by the device manufacturer, or product recall [2,3,12,13]. The true extent of the 3T device-associated *M. chimaera* infections has not yet been determined and it is likely to remain underestimated. Jointly with experts from various European countries, the European Centre for Disease Prevention and Control (ECDC) developed a clinical and environmental investigation protocol based on available experience [14]. Still, both clinical and environmental surveillance face technical challenges as (i) symptoms of invasive *M. chimaera* infection can occur more than 5 years after surgery, (ii) the clinical presentation is non-specific and can be indolent, (iii) diagnosis of *M. chimaera* infections by mycobacterial culture is slow and of low sensitivity unless infected tissue is obtained by invasive sampling, and (iv) identification of mycobacteria at the species level requires specialised DNA sequence-based testing. Thus far, no direct nucleic-acid amplification or metagenomics assay has been proposed for the rapid detection of *M. chimaera* in clinical or environmental samples.

An improved understanding of the risk determinants associated with the use of HCUs and the extent of the *M. chimaera* outbreak are critical for appropriate communication to healthcare providers and patients and for raising their awareness. Risk assessments at hospital level and the timely diagnosis and treatment of *M. chimaera* infection among exposed patients, as well as close collaboration between device manufacturers and regulatory agencies to ensure safe use of the HCUs are essential to close this patient safety loophole [2,12,15]. Further to this incident of contamination of devices during manufacturing, growing evidence of contamination of HCUs with diverse non-tuberculous mycobacteria and other opportunistic pathogens suggests a wider aerosol-borne infectious hazard from water-containing devices used in surgery that will require further risk assessment before and after putting such devices into clinical use [4,16].
