# Authors’ correction for Euro Surveill. 2016;21(46)

**DOI:** 10.2807/1560-7917.ES.2016.21.47.30404

**Published:** 2016-11-24

**Authors:** Eurosurveillance editorial team

**Affiliations:** 1 European Centre for Disease Prevention and Control (ECDC), Stockholm, Sweden

**Keywords:** Mycobacterium chimaera, waterborne infections, device-associated infections, patient safety

In the article ‘*Mycobacterium chimaera* infections associated with heater-cooler units (HCU): closing another loophole in patient safety’ by MJ Struelens et al., published on 17 November 2016, the following corrections were made on 21 November 2016, upon request of the authors:

The sentence ‘However, whole-genome sequence data from the outbreak investigations in Europe and the US have not been published to date.’ has been corrected to read ‘However, the full results of the analysis of whole-genome sequence data in relation to the epidemiological data from the outbreak investigations in Europe and the US have not been published to date.’

The sentence ‘To the best of our knowledge, the sharing before publication of preliminary genome sequence data on this emerging pathogen through public repositories, as advocated for improving public health investigations of international epidemics [9,10], has not yet been implemented.’ has been corrected to read ‘Of note, the sharing before publication of genome sequence data on this emerging pathogen through public repositories, as advocated for improving public health investigations of international epidemics [9,10], has been recently implemented by several investigators [7].’

